# Effects of nitrogen fertilization on protein and carbohydrate fractions of Marandu palisadegrass

**DOI:** 10.1038/s41598-021-94098-4

**Published:** 2021-07-20

**Authors:** Rhaony Gonçalves Leite, Abmael da Silva Cardoso, Natália Vilas Boas Fonseca, Maria Luisa Curvelo Silva, Luís Orlindo Tedeschi, Lutti Maneck Delevatti, Ana Cláudia Ruggieri, Ricardo Andrade Reis

**Affiliations:** 1 Department of Animal Sciences, São Paulo State University, Jaboticabal, SP 14884-900 Brazil; 2 Department of Animal Science, Texas A&M University, College Station, TX 77843-2471 USA

**Keywords:** Plant ecology, Ecology

## Abstract

The effects of nitrogen (N) fertilization levels on protein and carbohydrate fractions in Marandu palisadegrass pasture [*Urochloa brizantha* (Hochst. ex A. Rich.) R.D. Webster] were investigated in a pasture over five years. The experimental design was completely randomized with four levels of N (0, 90, 180, and 270 kg N ha^-1^, as urea) for five years, and with three replicates. The study was conducted in a continuously stocked pasture during the forage growing season (December to April) in a tropical region. The effects of N fertilization were similar across the five years. With increasing N fertilization, the concentrations of crude protein (CP) increased from 103 to 173 g kg^−1^ (*P* < 0.001), soluble fractions (Fraction A + B1) increased from 363 to 434 g kg^−1^ of total CP (*P* = 0.006); neutral detergent fiber (NDF) decreased from 609 to 556 g kg^−1^ (*P* = 0.037); indigestible NDF (*P* = 0.046), potentially degradable neutral detergent fiber (*P* = 0.037), and acid detergent fiber decreased (*P* = 0.05), and total digestible nutrient (TDN) increased (*P* < 0.001). Increasing N fertilization decreased the concentrations of Fraction C (*P* = 0.014) and total carbohydrates (*P* < 0.0001), and increased CP:organic matter digestibility (*P* < 0.01). Concentrations of neutral detergent fiber free of ash and protein (P = 0.003), indigestible neutral detergent fiber (*P* < 0.001), neutral detergent fiber potentially degradable (*P* = 0.11), CP (*P* < 0.001), Fraction A + B1 (*P* < 0.001), Fraction B2 (*P* < 0.001), Fraction B3 (*P* < 0.01), and non-structural carbohydrates differed (*P* < 0.001) across years. Therefore, N fertilization can be used to increase CP, soluble protein, and TDN.

## Introduction

Grassland areas are becoming increasingly important for animal production due to the increase in cattle production in Latin America, Africa, and Asia^1^. Areas in which tropical grasses predominate are known to have forage with low protein concentrations and digestibility^2^. This low nutritional value of tropical grasses could be due to poor pasture management (e.g., inappropriate pasture height), overgrazing, an absence or a low level of fertilization, and poor soil fertility^3^. Furthermore, feed and fodder scarcity is regarded as a general problem for low livestock productivity in tropical areas. However, adequate information regarding the nutritional value of grasses in terms to improve the profile of protein fractions with fertilization is lacking.

Nitrogen (N) fertilization can increase forage production and nutritional value in tropical regions^4^. N fertilizers can increase forage production and affect forage quality^4–8^. In grasslands, the concentration of forage protein, soluble carbohydrates, and cell wall components are essential because, when consumed by the animals, these are supplied to the rumen microbes and therefore affect animal maintenance and production^9–11^. Protein and carbohydrate concentrations of grasses and their digestibility are affected by fertilization, species, stage of maturity, management, and climatic factors^12–14^. These climatic factors e.g. precipation may vary along the year and therefore can affect the effect of N fertilization on nutritive value of grasses.

Particular fractions of proteins and carbohydrates are important; therefore, a system that classifies fractions based on their solubility has been proposed. The Cornell Net Carbohydrate and Protein System (CNCPS) was developed to account for nutrient fractionation. It was initially developed in a series of four papers^15–18^ and has received many updates and modifications^10,11^. The CNCPS is not the only mathematical model to adopt the fractionation of nutrients to improve diet evaluation^19^. In the CNCPS, the detergent fiber system of feed analysis is used to fractionate carbohydrates into fiber carbohydrates [neutral detergent fiber (NDF) and lignin] and non-fibrous carbohydrates (NFC; soluble sugars, starch, and pectin). Proteins are divided into fractions based on their fermentation characteristics (Fraction A = non-protein nitrogen, Fraction B1 = easily-degradable protein, Fraction B2 = intermediately-degradable protein, Fraction B3 = slowly-degradable protein, and Fraction C = non-degradable and unavailable to the animal), as described by Sniffen et al.^17^. Therefore, to improve the nutritional value of grasses, it is essential to increase CP and reduce fiber concentrations, and identify how the fractions are available for ruminal degradation and microbial growth. Tedeschi and Fox^10,11^ described additional modifications to the protein fractionation regarding the use of tungstic acid versus trichloroacetic acid to more consistently separate the non-protein fraction.

To apply the CNCPS to forages, it is necessary to characterize carbohydrates and N fractions^10,11^. Previous research has characterized protein fractions of various forage species from temperate areas^12,20^, including warm grasses [such as bermudagrass, stargrass, and bahiagrass]^21^ and guinea grass^22^. However, information related to the effects of N fertilization and annual changes in the carbohydrate and protein fractions of *Brachiaria* is limited. Furthermore, *Brachiaria* is the most utilized forage for cattle production in tropical areas, and the effects of N fertilization may vary among species and regions.

Thus, we studied the effect of N fertilization and year seasonality on carbohydrate and protein fractions of Marandu palisadegrass. We hypothesized that the concentrations of CP and Fraction A would increase with N fertilization level, whereas the fibrous carbohydrate fractions would decrease. Additionally, we hypothesized that the N fertilization effect on carbohydrates and protein fractions would vary with year.

## Results and discussion

In our studies with Marandu palisadegrass, a grazing management strategy with continuous stocking where 95% of the light is intercepted by the canopy resulted in forage at a height of 25 cm, a high green leaf proportion, and low amounts of dead material during the growing season^23,24^. The use of N fertilization^4^, different stocking rates, and supplementation^25–27^ are crucial for obtaining forage with a high nutritional value, resulting in a high weight gain per animal and area, and a reduction in slaughter age and greenhouse gas emissions. In the present study we did not find any interection N doses with years. Therefore, only the significative effects of N fertilization or variation within year are presented and discussed.

### Total and non-fibrous carbohydrates and total digestible nutrients

Total carbohydrate concentrations decreased linearly with increasing N levels (*P* < 0.01; Table 1). Nitrogen fertilization increases cell content concentrations (soluble fractions) and changed the sugar composition and bonds established between them in the cell wall^28^. Therefore, the reduction in TC represents the reductions observed in the NDF and ADF fractions (Table 1). The fibrous compounds in forage decrease with increasing N levels because this nutrient stimulate the growth of new tissues^29^. In the present study, we managed the pasture to reduce stem growth. A sward height of 25 cm stimulates tillering and growth of new tissues^24^.

**Table 1:** Average chemical composition for Marandu palisadegrass (g kg^-1^ dry matter) affected by nitrogen dose.

Variable	Nitrogen dose (kg N ha^-1^)				Effect^1^
	0	90	180	270	
TC	783.7 (15.6)	760.8 (16.0)	742.1 (15.4)	721.3 (17.8)	Linear
NFC	94.4 (4.5)	90.8 (5.2)	90.1 (6.6)	85.9 (8.1)	ns
apNDF	609.3 (7.0)	590.0 (6.9)	572.0 (5.7)	556.4 (5.6)	Linear
iNDF	187.8 (2.3)	177.1 (2.4)	174.1 (1.8)	156.5 (1.5)	Linear
NDFpd	421.5 (8.2)	413.0 (6.7)	397.9 (7.2)	399.9 (8.1)	Quadratic
ADF	327.0 (6.5)	307.0 (6.9)	301.6 (7.8)	294.7 (6.5)	Linear
Lignin	87.3 (2.1)	85.0 (2.4)	83.3 (2.5)	86.0 (1.9)	ns
TDN	629.9 (11.4)	636.0 (10.3)	638.5 (15.6)	642.2 (13.4)	Linear
CP:DOM (g CP kg^-1^ DOM)	125 (7)	152 (8)	174 (8)	195 (10)	Linear
CP	103.2 (10.1)	128.6 (11.3)	150.0 (14.4)	172.8 (14.3)	Linear
Fraction A + B1 (g kg^-1^ CP)	363.3 (8.2)	369.0 (7.2)	406.1 (8.1)	433.2 (7.2)	Linear
Fraction C (g kg^-1^ CP)	125.0 (3.5)	103.7 (2.8)	101.9 (3.7)	98.3 (2.9)	Linear

^1^Orthogonal polynomial effect of N doses. Effect probability (apNDF, *P* < 0.0001; iNDF, *P* = 0.046; NDFpd, *P* = 0.037; ADF, *P* = 0.05; Lignin, *P* = 0.19; TDN, *P* < 0.0001; TC, *P* < 0.0001; CP:DOM, *P* < 0.01; CP, *P* < 0.001; Fraction A + B1, *P* = 0.006; and Fraction C, *P* = 0.014). Within parentheses is the standard error of the means (SEM; ±).

**Figure 1:**
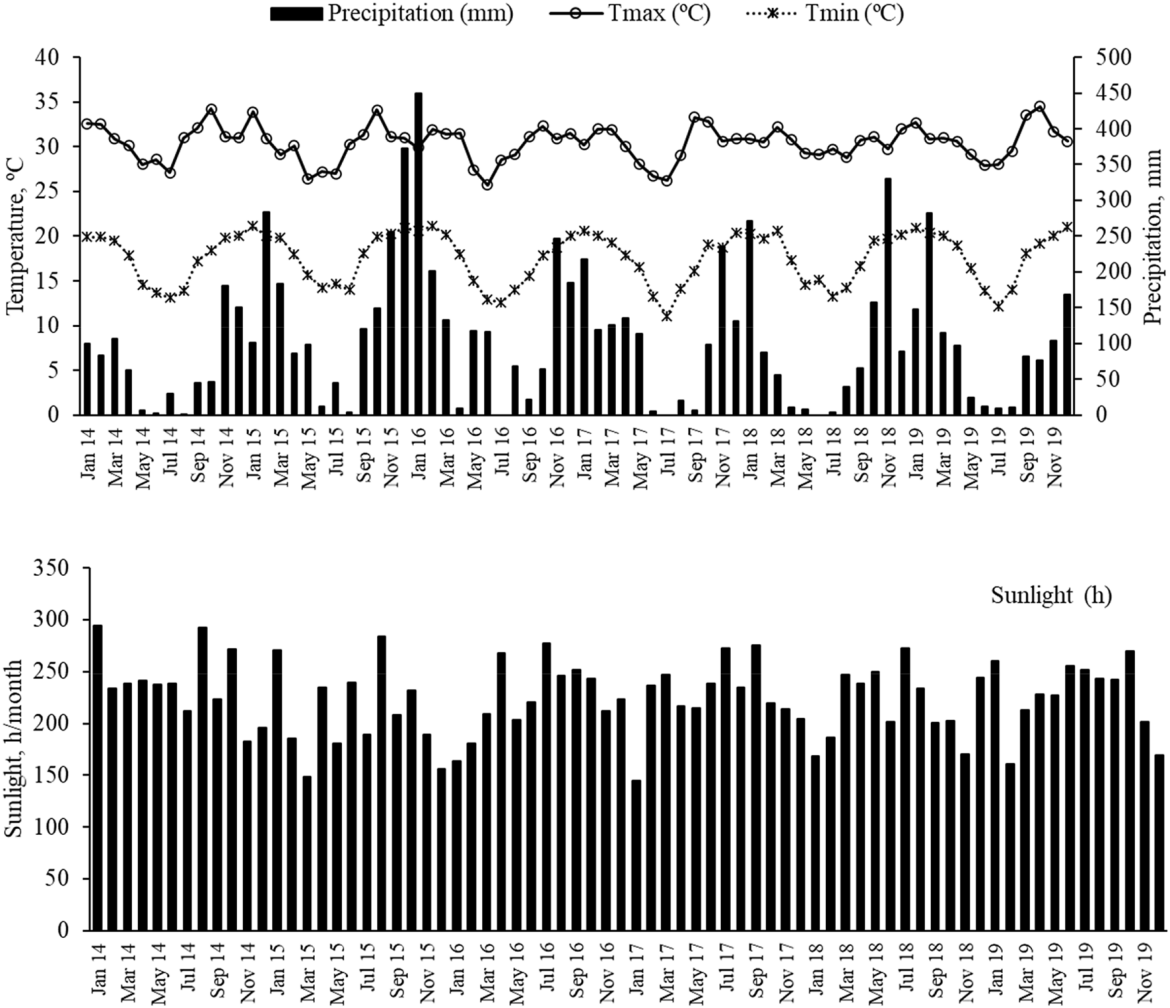
Precipitation, temperature, and sunlight from 2015 to 2019 at the experimental site at the São Paulo State University Jaboticabal, São Paulo, Brazil.

NFC varied according to the experimental year (*P* < 0.01; Table 2). The NFC fraction can be rapidly degraded in the rumen, and is necessary to maintain adequate carbohydrate and protein degradation synchrony, and promote adequate microbial growth^17^. Variations in climatic conditions, such as precipitation, alter the production of leaves and stems, and, consequently, cause changes in the concentrations of soluble sugars, starches, and pectins^22^. Our results are in agreeance with those of Santos et al.^22^, who observed high NFC when the highest precipitation occurred. Another climatic variable related to NFC is the sunlight, which affects the amount of glucose formed during photosynthesis^30^. Only the year affected NFC (Table 2). The highest NFC occurred in 2016, when the highest precipitation and lowest sunlight hours were recorded (Fig. 1 and Table 2). Greater sunlight increases the photosynthetic rate, which stimulates stem elongation by promoting cell growth. Cloudy and warm days stimulate the growth of new tissues^29^.

**Table 2:** Average chemical composition for Marandu palisadegrass (g kg^-1^ dry matter) affected by year.

Variable	Experimental year					Effect^1^
	2015	2016	2017	2018	2019	
apNDF	589.6 (5.4)	541.3 (5.7)	605.4 (6.7)	620.4 (4.9)	552.9 (4.7)	Cubic
iNDF	116.0 (1.0)	167.4 (6.0)	215.4 (7.6)	204.2 (4.6)	166.2 (9.6)	Quadratic
NDFpd	473.6 (3.9)	373.9 (7.1)	390.0 (5.6)	416.3 (5.2)	386.6 (3.4)	ns
ADF	298.2 (2.8)	254.4 (2.3)	302.7 (3.1)	361.5 (3.1)	312.3 (2.9)	Cubic
OM	911.5 (1.9)	922.0 (2.3)	919.0 (2.2)	911.9 (2.0)	908.6 (1.9)	Quadratic
NFC	136.3 (10.7)	246.6 (13.2)	162.5 (14.2)	134.2 (9.7)	170.6 (15.3)	Cubic
CP	123.1 (12.1)	130.1 (11.6)	127.5 (9.8)	142.2 (13.5)	170.2 (16.5)	Linear
Fraction A + B1 (g kg^-1^ CP)	325.4 (3.3)	302.1 (3.6)	367.9 (4.1)	382.9 (3.8)	586.0 (3.2)	Linear
Fraction B2 (g kg^-1^ CP)	282.5 (2.2)	245.3 (2.5)	284.0 (2.8)	177.8 (2.0)	166.6 (2.2)	Quadratic
Fraction B3 (g kg^-1^ CP)	314.0 (3.4)	340.4 (3.6)	229.6 (3.7)	264.5 (2.8)	196.1 (2.9)	Quadratic

^1^Orthogonal polynomial effect of N doses. Effect probability (apNDF, P = 0.003; iNDF, *P* < 0.001; NDFpd, *P* = 0.11; ADF, *P* < 0.0001; NFC, *P* < 0.001; CP, *P* < 0.001; Fraction A + B1, *P* < 0.001; Fraction B2, *P* < 0.001; and Fraction B3, *P* < 0.01). Within parentheses is the standard error of the means (SEM; ±).

Increasing N fertilization increased the TDN concentration (*P* < 0.01; Table 1). The TDN fraction is similar to OM in terms of digestibility and is usually found at a concentration of 60% in tropical grasses^28^. However, it increased on average from 62 to 65% in our study as N fertilization increased from 0 to 270 kg N ha^−1^. Increases in variables related to forage digestibility in response to N fertilization were also observed by several authors^7,21,22^. We found that TDN was strongly associated with NFC, NDFpd, and soluble protein (Tables 1 and 2).

### Neutral detergent fiber, indigestible detergent fiber, and potentially degradable detergent fiber

The concentration of NDF decreased linearly with increasing N levels (*P* < 0.001; Table 1). Conversely, there was a cubic relationship between NDF and year (*P* < 0.05; Table 2). Our results corroborate other studies that found a reduction in NDF with increasing N fertilization^4,7,21^. Nitrogen fertilization can increase cell content  and decrease the cell wall concentration^28^. However, this effect is climate-dependent, as observed in our study. Lower NDF concentrations occurred in 2016, when higher precipitation was observed (Fig. 1 and Table 2). Water availability in the soil is essential for N uptake by the plant. In the years that ocorred greater precipitation the conponents of cell wall descreased probably due increases in N recovery by the marandu palisade grass.

The iNDF concentration decreased linearly with increasing N fertilization (*P* < 0.01; Table 1). Few publications have reported iNDF concentrations. We presented this data because fiber is the most common variable used to predict the feed energy concentration, and there is a negative relationship between fiber concentration and available energy^31^. The reduction in iNDF concentration due to increasing N fertilization suggests that high N fertilization is a potental strategy to increase available energy in tropical grasses under grazing. However, this reduction was observed when the pasture was managed under a light interceptation level of 95%, which limited stem elogantion. Different results may occur in other forage management strategies^26^.

The N fertilization had a quadratic effect on NDFpd (*P* < 0.05; Table 2). The reduction of NDFpd with the application of N occurred up to a dose of 180 kg N ha^−1^. NDF provides energy for ruminal microbial syntheses, but also improves rumen function by adding structural carbohydrates to the ruminant diet^32^. The stabilization of the degradable fraction of NDF with increasing N fertilization suggests that the structural value, that is, the passage rate of ruminant diet, was maintained.

### Acid detergent fiber

Increasing N fertilization had a negative linear effect on ADF concentration (*P* < 0.01; Table 1). Previous research found that increasing N fertilization had little or no effect on ADF^7,29^. There was a quadratic relationship between ADF concentration and experimental year (*P* < 0.05; Table 2). The plant cell wall concentration can vary due to variations in precipitation and temperature. Rainfall and temperature are the major factors that affect plant maturity. The combination of relatively cooler temperatures and the absence of rain well into the growing season can result in a forage reproductive state, which could increase the fiber fractions^2^ and might explain these changes in ADF concentrations. The highest average ADF concentration (36.15%) was observed during the growing season of 2018 when lower precipitation occurred (Fig. 1).

### Total protein

A linear increase in CP concentration was observed with increasing N levels, and a linear relationship between CP and experimental year was observed (*P* < 0.001; Tables 1 and 2). Increases in CP concentrations of forage due to N fertilization are known. For example, Prine and Burton (1956) observed increases in CP as annual N fertilization levels increased in warm grasses. Similar results have been observed in other studies^4,7,8,29^. Additionally, we observed a cumulative effect of N fertilization on CP concentration over the duration of the experiment. Although forage CP concentrations can vary with precipitation and temperature^28^, the responses of CP to N fertilization observed could not be attributed to the climatic variables. The highest and lowest precipitation did not coincided with the experimental years that the highest and lowest CP concentrations occurred (Fig. 1 and Table 2).

The average CP concentration for Marandu palisadegrass is usually less than 10%^7,13,33^. The minimum value observed in our study was 12% (Tables 1 and 2). Our results are similar to those found previously in our experimental site (11%–14%; Barbero et al^25^; Koscheck et al.^27^) and that observed by McRoberts et al.^8^, which was 13%–15%. Nitrogen concentration, together with the cell wall concentration, is the most important factor in the supply of the required quantity of nutrients^26^. Our results suggest that five years of grazing at a fixed pasture height that corresponds to the 95% light interception during the growing season can result in high CP concentrations for Marandu palisadegrass, even without N fertilization. Therefore, a high animal performance could be obtained.

### Soluble protein: Fraction A and B1

The fraction of soluble protein increased linearly with increasing N levels (*P* < 0.0001; Table 1) and with experimental year (*P* < 0.01; Table 2). Previous research^8,21,22^ has indicated that increased N fertilization levels increase nitrate accumulation in the plant, which is a portion of Fraction A.

The soluble protein fraction varied from 325 to 586 g kg^−1^ CP throughout the experiment (Table 2). Several researchers have shown that the proportion of leaves and stems, leaf expansion rate, and tillering adapt to new grazing management targets^24,34^. In our study, the grazing management strategy allowed the growth of a greater proportion of green leaves, which probably explains the increase in soluble protein. High concentrations of Fraction A and B1 are desirable, as this fraction is rapidly degraded in the rumen and can result in greater animal performance^35^. Animal performance depends on microbial protein production that can be optimized with a greater amount of soluble N since the amount of energy does not limit microbial growth considering de NFC and NDFpd levels (Table 1).

### Fractions B2 and B3

We did not find any effect of increasing N fertilization on the protein fractions with moderate and low degradation rates. Conversely, these fractions varied quadratically with experimental year (*P* < 0.01; Table 2). Fraction B2 was approximately 40% lower during the fourth and fifth experimental years. The highest average value of Fraction B3 was observed in the year with the lowest precipitation (2016; Fig. 1). Fractions B2 and B3 are associated with membranes and extensins that are bound to hemicellulose and are dependent on the temperature. In our study, the temperature remained similar throughout the year, varying from 19 to 31 ºC (Fig. 1).

In contrast to our results, Fraction B2 has previously been found to vary with N fertilization. Rogers et al.^20^ found that Fraction B2 increased by approximately 35% in bermudagrass, and Johnson et al.^21^ observed an increase of up to 57% for warm-season grasses in Florida with N fertilization. Theses variations between theses studies and our findings is likely due to the pastures management differences. Berça et al.^14^ showed that pasture management play essential role in the variation of fiber concentration in marandu palisade grasss However, our results are in line with those of Santos et al.^22^, who only observed an effect of seasonality, with the highest values Fraction B2 being associated with low rainfall and high NDF.

### Protein fraction C: non-degradable nitrogen

Fraction C decreased linearly with N fertilization (*P* < 0.01; Table 1). This result differs from previous studies. Johnson et al.^22^ observed that Fraction C depends of the N fertilization dose for bahiagrass and stargrass being lower or higher to the lowest dose. Rogers et al.^20^ and Santos et al.^22^ did not find any effect of N fertilization on Fraction C. However, similar to our results, Zhang et al.^36^ found a decrease in Fraction C by 55.3% in annual ryegrass forage with increasing N fertilization.

Fraction C corresponds to N linked with lignin, tannin-protein complexes, and Maillard products, which are highly resistant to enzymes produced by the microbes in the rumen, being considered unavailable to the animal^10,11,17^. Increasing N fertilization improved protein availability in Marandu palisadegrass. Growing Marandu palisadegrass under a grazing management strategy with a pasture height corresponding to a light interception of 95% resulted in a low concentration of structural carbohydrates associated with lignin and a low concentration of Fraction C^25,27^.

The crude protein:organic matter digestibility ratio increased linearly in response to N fertilization (Table 1). According to Poppi and McLennan^37^, a CP:DOM of 160 g resulted in high efficiency of microbial growth; however, CP:DOM values above 210 g caused high N losses. Maximum N utilization efficiency values in post-weaning beef cattle reared in tropical grass pasture were observed below 200 g of CP/kg DOM, and losses occurred above 200 g of CP/kg DOM^38^. In the present study values above 160 g of CP/ kg DOM were observed at doses of 180 and 270 kg of N/ha.

### Conclusions

Estimations of carbohydrate and protein fractions can increase the nutrient utilization efficiency and determine the type of supplementation needed under each pasture management strategy. We observed an increase in CP and soluble protein with increasing N fertilization, leading to less protein being required in supplements. Therefore, it is necessary to include soluble carbohydrates, starch, and pectin in the diet to maintain protein and carbohydrate degradation synchrony in the rumen and optimize microbial growth. We observed higher CP, soluble protein, and TDN concentrations when 90 kg N ha^−1^ was applied, suggesting that this dose is the most suitable for Marandu palisadegrass under a continyos stocking and a pasture management strategy with canopy target of 25 cm during the growing season. Future studies should be directed toward understanding undegradable protein supplementation, inclusion of proteins from legumes, and high degradable carbohydrate fractions in tropical diets.

## Methods

### Experimental area and design

We conducted a five-year experiment in the Forage and Grasslands Laboratory of São Paulo State University, “Julio de Mesquita Filho” (UNESP) (Jaboticabal, São Paulo, Brazil), during the summer growing seasons (December to April) of 2014/2015, 2015/2016, 2016/2017, 2017/2018, and 2018/2019. The climate of the experimental area is classified as a subtropical humid climate, with wet summers and dry winters^4^. The mean annual rainfall is 1424 mm, the mean air temperature is 22.3 °C, and the soil is a Rhodic Ferralsol derived from basalt^39^. The pasture was established in 2001 with *Urochloa brizantha* (Hochst ex A. Rich) Stapf Marandu (Marandu palisadegrass). The precipitation and temperature in the experimental area are presented in Fig. 1. During the growing season, the average minimum and maximum temperatures were 19 ºC and 31 ºC, respectively, and the average monthly sunlight varied from 150 to 300 h. Mean soil chemical characteristics evaluated always on the September from 2015 to 2019 are presented in Table 3. In November of each year, maintenance fertilizer was applied to all paddocks at 50 kg P_2_O_5_ (superfosfate) and 70 kg K_2_O (potassium chloride) per hectare. Soil texture content are 291, 123, 588 g kg^−1^ soil of clay, silte and sand, respectively.

**Table 3:** Means soil chemical caracteristcs of the experimental area at the depth of 0 – 20 cm, Jaboticabal– SP.

Year	P resin	S-SO_4_^2-^	OM	pH CaCl_2_	K^+^	Ca^2+^	Mg^2+^	H^+^Al	Al^3+^	CEC	V%
	– mg/dm^3^–	g/dm^3^		––mmolc/dm^3^––							
2015	12	16	25	5,2	2,6	36	11	22	0	92	62
2016	15	11	27	5,1	2,8	37	17	28	0	98	61
2017	13	12	28	5,1	3,0	35	14	24	0	95	59
2018	12	16	26	5,2	3,0	34	13	22	0	94	59
2019	11	15	28	5,2	2,9	32	13	23	0	92	57

P = phospuros, S-SO_4_^2-^ = sulfate, OM = organic matter, K = potassium, Ca = Calcium, Mg = magnesium, Al = alluminum, CEC = capacity os exchange cations and V% = bases saturation.

The experiment consisted of four nitrogen doses (0, 90, 180, and 270 kg N ha^−1^) in a completely randomized design with three replicates per treatment, totaling 12 paddocks (experimental units). The paddock areas were 1.3 ha, 1 ha, 0.7 ha, and 0.5 ha for the treatments 0, 90, 180, and 270 kg N ha^−1^, respectively. The experimental area included a reserve area of 3 ha for the spare animals. The experimental area meets the criterion of soil homogeneity to conduct a CRD experiment. The source of N was urea, and its application was split across three times during the rainy season (Begin of December, end of January and begin of March). Animals used in research were cared for according to the rules of the São Paulo State University Animal Care and Use Committee and the National Council of Animal Experimentation Control. The committee reviewed and approved the experiment and all procedures carried out in the study (Certificate number 12703/15). We declare that no permissions or specific requirement to collect, analyse and work with *Urochloa brizantha* are requeride by local and national Brazilian authorities.

### Grazing management

The present study was conducted under grazing conditions. Each year, 72 young Nellore bulls (*Bos indicus*) were used to measure animal productivity (average daily gain and gain per ha). The bulls had an initial body weight (mean ± standard deviation) of 352 ± 5 kg, 334 ± 2 kg, 315 ± 6 kg, 220 ± 2 kg, and 206 ± 9 kg in the first, second, third, fourth, and fifth experimental years, respectively.

The experimental units were grazed with a continuous and variable stocking rate^40^. To maintain the pasture height, the stocking rate was adjusted weekly. The grazing target was a pasture height of 25 cm. At this height, the canopy intercepted 95% of the incident light under our experimental conditions. At this light interception, the maximal net forage accumulation is achieved, resulting in high average daily gain and gain per hectare^24,25,27^.

### Forage collection and preparation

Forage samples were harvested at 28-d intervals beginning in the middle of December from 20 points per hectare using the hand-plucking method^41^. In the hand-plucked method the grazing behavior of the animal is firstily observed ; then herbage samples are taken manualy mimicking the animal foraging. Approximately 200 g of fresh matter was harvested per sample to determine forage chemical compositions. Samples were dried in a forced-air oven (55 ± 5 °C, for 72 h), ground in a mill through a 2-mm screen (Thomas-Wiley Laboratory Mill Model 4, H. Thomas Co.), and taken to the laboratory for analysis.

### Chemical analysis

Laboratory analyses included measurements of dry matter (DM), organic matter (OM), and ash determined using the following procedures from AOAC^42^: AOAC 934.01 for DM, AOAC 942.05 for OM, and AOAC 942.05 for ash. Crude protein (CP) concentration (AOAC 990.03) was estimated using a LECO FP 528 device (Leco Corporation, Michigan, USA). Neutral detergent fiber (NDF) and acid detergent fiber (ADF) were determined using the procedures described by ANKOM Technology^43^.

The indigestible neutral detergent fiber (iNDF) was quantified by in situ incubation of hand-plucked samples conditioned in ANKOM brand F-57 filter bags arranged in the rumen of fistulated animals for 288 h^44^. After being thoroughly washed, oven-dried at 55 °C for 72 h, and then dried in an unventilated oven at 105 °C for 45 min, the bags were weighed to obtain the indigestible DM. Subsequently, the bags were subjected to NDF quantification using the ANKOM fiber analyzer, as cited above.

Fractions of forage carbohydrates were obtained following Sniffen et al.^17^ for total carbohydrates (TC) and non-fibrous carbohydrates (NFC). Total digestible nutrient (TDN) was calculated using the equation of energy using multiple components^31^. The potentially degradable NDF (NDFpd) was calculated by subtracting iNDF from the NDF.

The fractionation of protein was determined as described by Sniffen et al.^17^. Fraction A was obtained by extracting the soluble N using trichloroacetic acid^45^ and calculating the difference between the total N concentration and the non-protein nitrogen^17^. For quantification of buffer soluble protein (Fractions A and B1), 0.50 g of sample was extracted with 50 mL of borate-phosphate buffer and 1 mL of sodium azide solution. Fraction B2 was calculated by subtracting Fraction A, Fraction B1, and the neutral detergent insoluble protein (N-NDF) from the total N. Fraction B3 was the difference between the N-NDF and the acid detergent insoluble protein (N-ADF). Fraction C was the N-ADF^17^. All fractions were expressed and g kg^-1^ CP.

### Statistical analysis

Data were analyzed using the LME function of R for mixed models (package NLME, R version 3.4.5). The statistical model included N level, year, and their interaction as fixed effects, and paddock was a random effect. All variables were analyzed as repeated measures. The best covariance structure used for repeated measures was chosen as the one that achieved the lowest corrected Akaike or Bayesian information criteria. The statistical model was:

γijk = µ + αi + πj(i) + βk + αβik + εijk, where: γijk is the observation, µ is the overall mean, α is the fixed effect of N doses, πj(i) is the random error associated with the N doses and repetitions, βk is the fixed effect of year (time), αβik is the N doses by year interaction and εijk equals the residuals error.

When a significant effect was found, orthogonal polynomial contrasts were performed to assess the effects of N level and year on the variables.

## Data Availability

Data will be made available upon request for authors.
